# Endothelial and Smooth Muscle Cells from Abdominal Aortic Aneurysm Have Increased Oxidative Stress and Telomere Attrition

**DOI:** 10.1371/journal.pone.0035312

**Published:** 2012-04-13

**Authors:** Giuseppe Cafueri, Federica Parodi, Angela Pistorio, Maria Bertolotto, Francesco Ventura, Claudio Gambini, Paolo Bianco, Franco Dallegri, Vito Pistoia, Annalisa Pezzolo, Domenico Palombo

**Affiliations:** 1 Department of Surgical Sciences and Integrated Diagnostics, University of Genoa, Genoa, Italy; 2 Laboratory of Oncology, IRCCS Gaslini Hospital, Genoa, Italy; 3 Epidemiology and Biostatistics Unit, IRCCS Gaslini Hospital, Genoa, Italy; 4 Department of Internal Medicine, University of Genoa, Genoa, Italy; 5 Department of Legal Medicine, University of Genoa, Genoa, Italy; 6 Laboratory of Pathology, IRCCS Gaslini Hospital, Genoa, Italy; 7 Department of Molecular Medicine, Sapienza University of Rome, Rome, Italy; Maastricht University, The Netherlands

## Abstract

**Background:**

Abdominal aortic aneurysm (AAA) is a complex multi-factorial disease with life-threatening complications. AAA is typically asymptomatic and its rupture is associated with high mortality rate. Both environmental and genetic risk factors are involved in AAA pathogenesis. Aim of this study was to investigate telomere length (TL) and oxidative DNA damage in paired blood lymphocytes, aortic endothelial cells (EC), vascular smooth muscle cells (VSMC), and epidermal cells from patients with AAA in comparison with matched controls.

**Methods:**

TL was assessed using a modification of quantitative (Q)-FISH in combination with immunofluorescence for CD31 or α-smooth muscle actin to detect EC and VSMC, respectively. Oxidative DNA damage was investigated by immunofluorescence staining for 7, 8-dihydro-8-oxo-2′-deoxyguanosine (8-oxo-dG).

**Results and Conclusions:**

Telomeres were found to be significantly shortened in EC, VSMC, keratinocytes and blood lymphocytes from AAA patients compared to matched controls. 8-oxo-dG immunoreactivity, indicative of oxidative DNA damage, was detected at higher levels in all of the above cell types from AAA patients compared to matched controls. Increased DNA double strand breaks were detected in AAA patients *vs* controls by nuclear staining for γ-H2AX histone. There was statistically significant inverse correlation between TL and accumulation of oxidative DNA damage in blood lymphocytes from AAA patients. This study shows for the first time that EC and VSMC from AAA have shortened telomeres and oxidative DNA damage. Similar findings were obtained with circulating lymphocytes and keratinocytes, indicating the systemic nature of the disease. Potential translational implications of these findings are discussed.

## Introduction

The human telomere is a simple repeating sequence of six bases, TTAGGG, located at the ends of chromosomes [1]–[3]. Normal human diploid cells possess limited capacity for proliferation in culture and this finite replicative lifespan has frequently been used as model of human aging in mitotic tissues and organs [4]–[6]. Each replicative cycle is associated with progressive reduction in telomere length (TL). So TL is an effective indicator of the number of cell divisions undergone. When telomere shortening reaches a critical threshold, cell senescence is triggered [7]–[12]. Telomere shortening may be accelerated by iatrogenic (e.g. telomere shortening occurs after bone marrow transplantation) or environmental factors (e.g. oxidative stress, inflammation and smoke) [13]–[15].

AAA is a complex multi-factorial disease with life-threatening complications and is characterized by a progressive enlargement of the infra-renal abdominal aorta, spontaneously evolving toward rupture [16]–[22]. Aneurysms typically have no signs or symptoms, and rupture of AAA has a high mortality rate. The current management strategy for patients with AAA includes a combination of anatomical imaging, watchful waiting and surgical intervention to prevent deadly ruptures. Decision to intervene surgically depends on the size and location of the AAA [16]–[22]. The timing of therapy and imaging is difficult but crucial, since both invasive repair and progressive disease carry significant risks. Smaller AAA may rupture between successive scheduled imaging sessions, while some large but relatively stable AAA are treated surgically, exposing patients to unnecessary risks. Multiple environmental and genetic risk factors are involved in AAA formation and progression [16]. AAA is typically a disease of adulthood [17], but it can develop also in childhood in association with Marfan, Ehlers-Danlos, and Loeys-Dietz genetic syndromes [18]. Furthermore, AAA has been reported in children with X-linked immunodeficiency and Wiskott-Aldrich syndrome [19]. It has been demonstrated that mutations in *ACTA2* gene, coding smooth muscle α-actin, and in *MYH11* gene, coding smooth muscle myosin heavy chain, are responsible for 14% of inherited ascending thoracic aortic aneurysms and dissections (TAAD) [20], [21]. Genome wide association studies have led to the identification of common sequence variants on chromosomes 19q13, 4q31, 9q33 and 9p21 predisposing to AAA in non-syndromic individuals [22]–[24].

Insight into the pathobiology of AAA is evolving quickly, and increasing evidence points to an important role for innate immune cells [25], [26]. Monocytes/macrophages infiltrate the vessel wall and release proteases, among them elastase and metalloproteinases, that compromise the integrity of the vascular wall through degradation of the extracellular matrix [27]–[31]. Monocytes/macrophages also secrete inflammatory cytokines in the media and adventitia of aneurysmatic vessels, such as TNFα, IFNγ and IL-6 [27]–[31]. Neutrophils produce large amounts of proteases, and recent experimental studies have pointed out the determinant role of neutrophils in AAA development [27], [28]. The inflammatory process leads to protease-mediated degradation of the extracellular matrix and apoptosis of vascular smooth muscle cells (VSMC), which are the predominant matrix synthesizing cells of the vascular wall [32]. These processes act in concert to progressively weaken the aortic wall resulting in dilation and aneurysm formation. Oxidative stress, that can contribute to inflammation, has been shown to be involved in AAA pathogenesis [32]–[37]. There is, therefore, a need for better risk prediction in order to personalize strategies for the individual patient. Recent studies suggest that biological ageing of the vasculature may play a role in AAA pathogenesis [38], [39]. Thus, TL in circulating blood lymphocytes from AAA patients was found to reflect that in aortic wall, suggesting that lymphocytes DNA content may represent a surrogate marker of vascular ageing [38], [39]. A limitation of the latter study deals with the lack of information about the cell types undergoing telomere shortening in the aortic wall from AAA patients.

In this study, we explored TL in endothelial cells (EC) and VSMC from aortic segments in close proximity to aneurysm, as well as in blood lymphocytes and epidermal cells, from AAA patients and concomitantly studied oxidative stress in the same cell types. The results obtained demonstrate for the first time that EC and VSMC from patients with AAA displayed shorter TL and markers of augmented oxidative stress.

## Results

### Telomere length in endothelial cells, vascular smooth muscle cells, blood lymphocytes and epidermal cells from patients with abdominal aortic aneurysms (AAA)

**Figure 1:**
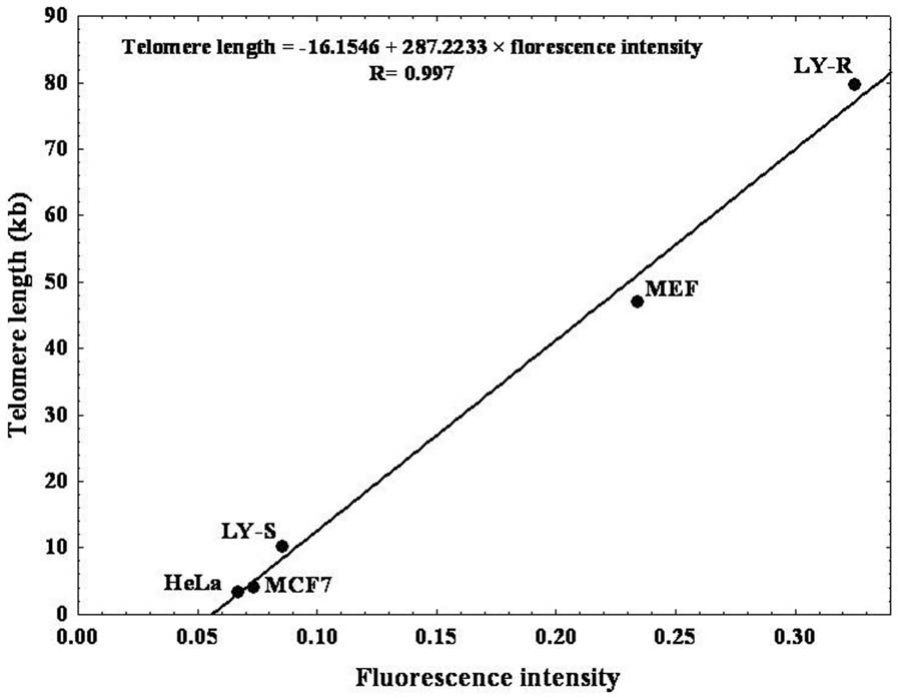
Calibration of quantitative FISH analysis. Telomere fluorescence values (TFU), obtained after hybridization with Cy3 pan-telomeric probe, are converted into kb by external calibration with the L5178Y-S and L5178Y-R murine lymphoma cell lines, MEF murine fibroblast cell line, MCF7 and HeLa human tumor cell lines with known TL of 10.2 Kb, 79.7 Kb, 47 Kb, 4.07 Kb, and 3.44 Kb respectively. Fluorescence intensity (TFU) plotted against the size of TTAGGG repeats sequences (Kb) of the five tumor cell lines. The resulting calibration line was used to transform arbitrary fluorescence intensity units into telomere length in Kb by means of the formula Y = −16.1546+287.2233×X (R = 0.997).

Telomere length (TL) was measured using quantitative fluorescence *in situ* hybridization (Q-FISH) [40]–[43] the only technique suitable to perform this analysis in individual cells. The telomere hybridization fluorescence intensities were expressed in arbitrary telomere fluorescence units (TFU) (Table 1). We have here developed a new modification of conventional Q-FISH allowing to quantify telomere repeat length in kilobase (Kb) by calibration with a panel of five tumor cell lines of known TL (Fig. 1). The resulting calibration line was used to express arbitrary TFU in Kb only for FISH performed in cultured or cytospined cells, but not in paraffin-embedded tissue.

**Table 1:** Median telomere fluorescence.

	Lymphocytes (TFU)	Endothelial cells (TFU)	Smooth muscle cells (TFU)	Epidermal cells (TFU)
**Patient 1**	0.10390	0.10110	0.09960	-
**Patient 2**	0.10325	0.09570	0.09465	-
**Patient 3**	0.09955	0.09950	0.09670	-
**Patient 4**	0.09710	0.09730	0.09345	-
**Patient 5**	0.09365	0.08975	0.09485	-
**Patient 6**	0.09475	0.09515	0.09675	-
**Patient 7**	0.10215	0.10170	0.10035	-
**Patient 8**	0.09240	0.09375	0.09435	-
**Patient 9**	0.09200	0.08885	0.09365	-
**Patient 10**	0.08985	0.09435	0.09110	0.09105
**Patient 11**	0.09230	0.09660	0.09925	0.09555
**Patient 12**	0.09695	0.09615	0.09060	0.09640
**Patient 13**	0.09230	0.09790	0.10075	-
**Patient 14**	0.09985	0.09565	0.09655	0.10010
**Patient 15**	0.09910	0.09985	0.10040	0.10050
**Patient 16**	0.10070	0.10190	0.09750	0.09975
**Patient 17**	0.10435	0.09240	0.09380	0.09405
**Patient 18**	0.09020	0.10450	0.10350	0.10360
**Patient 19**	0.09905	0.10165	0.10290	0.10075
**Patient 20**	0.10425	0.09275	0.09590	0.09845
**Patient 21**	0.10115	0.09860	0.09610	0.09785
**Patient 22**	0.09940	-	-	-
**Patient 23**	0.09375	-	-	-
**Control 1**	0.11300	0.10190	0.10265	0.10195
**Control 2**	0.11100	0.10420	0.10700	0.10120
**Control 3**	0.10700	0.10285	0.10355	0.10325
**Control 4**	0.10565	0.10135	0.10340	0.10240
**Control 5**	0.11240	0.10425	0.10600	0.10190
**Control 6**	0.10385	0.10330	0.10375	0.10065
**Control 7**	0.10840	0.10235	0.10325	-
**Control 8**	0.10905	0.10300	0.10615	-
**Control 9**	0.10490	0.10270	0.10365	-
**Control 10**	0.11220	0.10330	0.10695	-
**Control 11**	0.10655	0.10335	0.10560	-
**Control 12**	0.10565	0.10295	0.10665	-
**Control 13**	0.10380	0.10305	0.10595	-
**Control 14**	0.10165	0.10285	0.10570	-
**Control 15**	0.11445	0.10300	0.10485	-
**Control 16**	0.11425	0.10270	0.10660	-
**Control 17**	0.10190	0.10320	0.10552	-
**Control 18**	0.10470	0.10255	0.10650	-
**Control 19**	0.10325	0.10305	0.10715	-
**Control 20**	0.09825	0.10315	0.10565	-
**Control 21**	0.11515	-	-	-
**Control 22**	0.10725	-	-	-
**Control 23**	0.10990	-	-	-
**Control 24**	0.10210	-	-	-
**Control 25**	0.11110	-	-	-
**Control 26**	0.10425	-	-	-
**Control 27**	0.10855	-	-	-
**Control 28**	0.10725	-	-	-
**Control 29**	0.09750	-	-	-
**Control 30**	0.09960	-	-	-
**Control 31**	0.10340	-	-	-
**Control 32**	0.10465	-	-	-
**Control 33**	0.10665	-	-	-
**Control 34**	0.10090	-	-	-

A minimum of 20 nuclei were scanned for every sample and the mean value of the fluorescence ratios of all cells analyzed was calculated.

**Figure 2:**
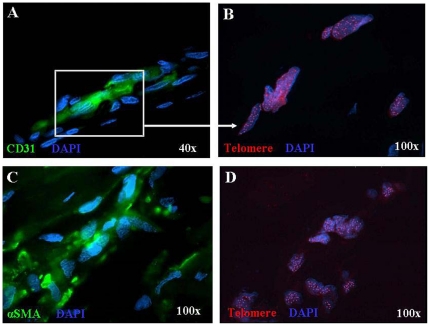
Telomere length of EC and VSMC from patients with AAA measured using Q-FISH and immunofluorescence. **A**) Aortic aneurysmatic wall derived EC stained with anti-CD31 mAb (green). The inset shows the nuclei analyzed by Q-FISH. **B**) EC interphase nuclei hybridized with Cy3-PNA telomeric probe (red signals). **C**) Aortic aneurysmatic wall derived VSMC stained with anti-α-smooth muscle actin mAb (green). **D**) VSMC nuclei hybridized with Cy3-PNA telomeric probe (red signals). DAPI was used to label nuclei.

**Figure 3:**
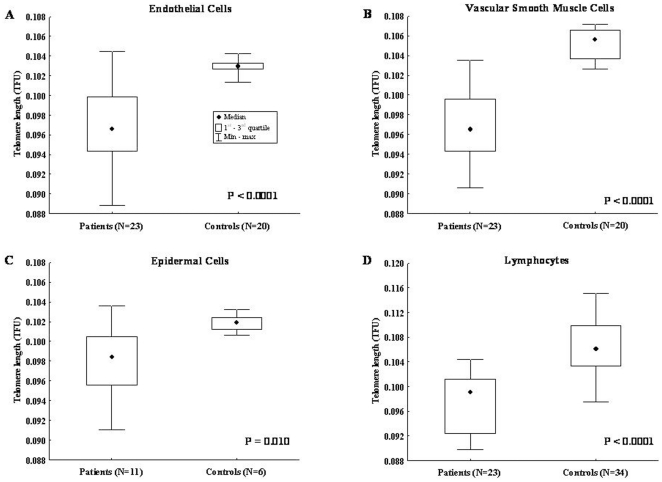
Telomere length of EC, VSMC, blood lymphocytes and epidermal cells from patients with AAA and controls. **A**) Telomeres length in EC from 21 AAA patients and in EC from 20 normal aorta. **B**) Telomeres length in VSMC from 21 AAA patients and in VSMC from 20 normal aorta. **C**) Telomeres length in epidermal cells from patients with 11 AAA and in these same cells from 6 controls. **D**) Telomeres length in peripheral blood lymphocytes in 23 AAA patients and in 34 controls.

We first investigated TL in aortic tissue from AAA patients and controls. Serial tissue sections were stained with CD31 and anti α-SMA mAbs by immunofluorescence in order to identify EC and VSMC, respectively (Fig. 2, A and C), and tested for TL in these cell types by Q-FISH (Fig. 2, B and D). Telomeres were significantly shorter in EC from AAA patients (median: 0.097 TFU; 1^st^–3^rd^ q: 0,094–0.100) than in EC from normal aorta (median: 0.103 TFU; 1^st^–3^rd^ q: 0.102–0.103) (p<0.0001) (Fig. 3 A). Likewise, telomeres were significantly shorter in VSMC from AAA patients (median: 0.097 TFU; 1^st^–3^rd^ q: 0.094–0.100) than in VSMC from normal aorta (median: 0.106 TFU; 1^st^–3^rd^ q: 0.104–0.107) (p<0.0001) (Fig. 3 B).

Subsequent experiments were performed measuring TL in epidermal cells from skin biopsies tested as control cells not involved in vascular lesions. Q-FISH revealed that telomeres in epidermal cells from patients with AAA were significantly shorter (median: 0.098 TFU; 1^st^–3^rd^ q: 0.096–0.100) than in the same cells from controls (median: 0.102 TFU; 1^st^–3^rd^ q: 0.101–0.102) (p = 0.010) (Fig. 3 C).

We next analyzed TL in peripheral blood lymphocytes from 23 AAA patients and 34 age-matched healthy donors (Fig. 3 D). Telomeres were significantly shorter in AAA patients (median: 0.099 TFU; 1^st^–3^rd^ q: 0.092–0.101) than controls (median: 0.106 TFU; 1^st^–3^rd^ q: 0.103–0.109) (p<0.0001) (Fig. 3 D) and this difference remained significant after adjusting for age (p<0.0001).

**Figure 4:**
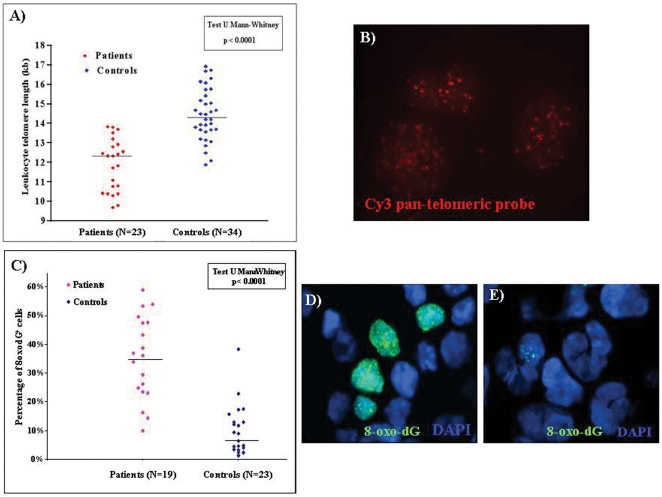
Telomere length and oxidative DNA damage in lymphocytes from each individual AAA patient and control. **A**) Telomere length of peripheral blood lymphocytes from 23 patients with abdominal aortic aneurysms (AAA), and 34 healthy donors. Bars represent the mean. **B**) Lymphocytes interphase nuclei from an AAA patient hybridized with Cy3-PNA telomeric probe (red signals). **C**) Oxidative DNA damage in peripheral blood lymphocytes from 19 patients with AAA, and 23 healthy donors. **D**) A representative immunostaining of anti-8-oxo-dG (green) of blood lymphocytes from an AAA patient. Arrows show nuclei intensively staining for 8-oxo-dG (green). **D**) Immunostaining of anti-8-oxo-dG (green) of blood lymphocytes from an healthy donor. Arrow shows nucleus with several small positive regions.

Then the calibration line was used to convert telomere fluorescence intensity in Kb for each lymphocyte sample. There was a median difference of 2 Kb in TL between lymphocytes from AAA patients and control group. Fig. 4 A shows TL in lymphocytes from each individual AAA patient and control tested, while Fig. 4 B shows a representative experiment in which patient lymphocytes were hybridized to a pan-telomeric probe.

In AAA patients, no correlation was found between blood lymphocyte TL and age (r = 0.56). Subjects with a positive family history of AAA had a significantly shorter mean blood lymphocyte TL compared to individuals without a family history of AAA. Positive family history of AAA was the only risk factor among those tested (aneurism size, smoking, hypercholesterolemia, hypertriglyceridemia, hypertension, body mass index) to be significantly associated with short lymphocyte TL (p = 0.012).

We next investigated correlations among TL of blood lymphocytes, EC, VSMC and epidermal cells from AAA patients. A strong correlation was identified between lymphocytes and epidermal cells (r_S_ = 0.84), lymphocytes and EC (r_S_ = 0.80), VSMC and epidermal cells (r_S_ = 0.80), lymphocytes and VSMC (r_S_ = 0.74), EC and VSMC (r_S_ = 0.72), and EC and epidermal cells (r_S_ = 0.72).

Taken together, these results demonstrate unambiguously that telomere shortening in AAA patients is a systemic phenomenon involving different cell types in different anatomical locations.

### Oxidative DNA damage in endothelial cells and vascular smooth muscle cells from aortic aneurysmatic wall

ROS, being products of normal cellular metabolism, exert a substantial influence on cell senescence, partly related to their ability to react with DNA [44]. ROS production may be assessed using antibodies against the specific “footprints” of oxidative damage. 7, 8-dihydro-8-oxo-2′-deoxyguanosine (8-oxo-dG) is a commonly used marker of oxidative stress-derived DNA damage after ROS attack [45], and can be measured by immunofluorescence.

**Figure 5:**
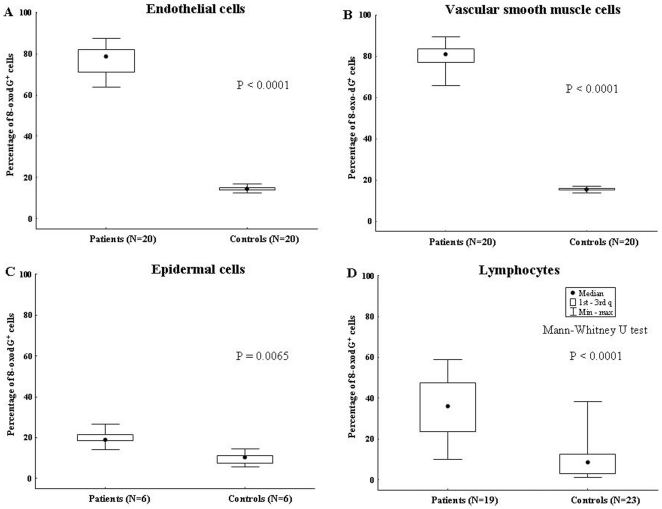
Oxidative DNA damage of EC, VSMC, blood lymphocytes and epidermal cells from patients with AAA and controls. **A**) The percentage of 8-oxo-dG^+^ nuclei in EC from 20 AAA patients and in EC from 20 normal aorta. **B**) The percentage of 8-oxo-dG^+^ nuclei in VSMC from 20 AAA patients compared to VSMC from 20 normal aorta. **C**) 8-oxo-dG^+^ nuclei in epidermal cells from 6 AAA patients and in the same cell type from 6 controls. **D**) The percentage of 8-oxo-dG^+^ nuclei in peripheral blood lymphocytes from 19 AAA patients compared to 23 controls.

Fig. 4 C shows the percentage of 8-oxo-dG^+^ lymphocytes from each individual AAA patient and control tested. Fig. 4 D shows a representative nuclear staining of lymphocytes from an AAA patient, while Fig. 4 E shows 8-oxo-dG^+^ blood lymphocytes from an healthy donor displaying only occasional low level staining. We found that the percentage of 8-oxo-dG^+^ nuclei in peripheral blood lymphocytes was significantly augmented in AAA patients (n = 19, median: 36.1%; 1^st^–3^rd^ q: 24.2–47.6) compared to controls (n = 23, median: 8.5%; 1^st^–3^rd^ q: 3.2–12.8) (p<0.0001) (Fig. 5). We next stained aortic tissue sections from AAA patients with anti-8-oxo-dG mAb and found significantly higher percentage of 8-oxo-dG^+^ nuclei in EC from AAA patients (median: 78.8%; 1^st^–3^rd^ q: 71.1–82.1) than in EC from normal aorta (median: 14.4%; 1^st^–3^rd^ q: 13.7–15.2) (p<0.0001) (Fig. 5 A). Furthermore, the percentage of 8-oxo-dG^+^ nuclei was significantly increased in VSMC from AAA patients (median: 81.1%; 1^st^–3^rd^ q: 77.2–83.6) compared to VSMC from normal aorta (median: 15.4%; 1^st^–3^rd^ q: 14.5–16.1) (p<0.0001) (Fig. 5 B).

Finally, 8-oxo-dG^+^ nuclei in epidermal cells from AAA patients were significantly more abundant (median: 19.1%; 1^st^–3^rd^ q: 18.5–20.9) than in the same cells from controls (median: 10.5%; 1^st^–3^rd^ q: 8–11.2) (p = 0.0065) (Fig. 5 C).

Taken together, these experiments demonstrated that, in analogy to that observed for TL, oxidative DNA damage was significantly higher in EC, VMSC, epidermal cells and lymphocytes from AAA patients than in the corresponding normal cellular counterparts.

### Focal expression of phosphorylated histone H2AX (γH2AX) in endothelial cells and vascular smooth muscle cells from aortic aneurysmatic wall

The histone protein H2AX is a central component of numerous signaling pathways in response to DNA double-strand breaks (DSBs), which becomes rapidly phosphorylated to form γH2AX at nascent DSB sites [46]. Shortened telomeres fail to protect the end of chromosomes. The uncovered DNA double-stranded end induces formation of γH2AX foci that represent excellent markers of telomere erosion and hence replicative senescence [47].

**Figure 6:**
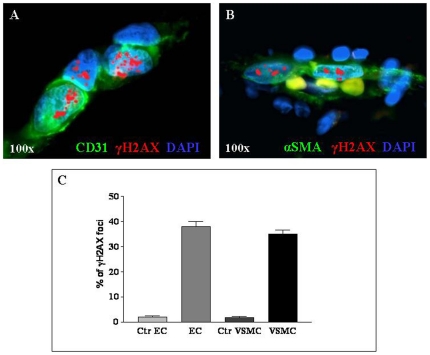
γH2AX staining patterns observed in EC and VSMC from patients with AAA. **A**) Aortic aneurysmatic wall derived EC stained with anti-CD31 (green) and anti-γH2AX (red) mAbs. **B**) Aortic aneurysmatic wall derived VSMC stained with anti-α-smooth muscle actin (green) and anti-γH2AX (red) mAbs. Focal staining for γH2AX is evident (A, B). Nuclei stained with DAPI (A, B). Magnification, 100× (A, B). **C**) γH2AX-positive foci were significantly more abundant in EC and VSMC from AAA wall compared to controls (p = 0.006 and p = 0.010 respectively).

We next investigated by immunofluorescence γH2AX expression in EC and VSMC from AAA *vs* control aortic walls. EC and VSMA were detected by staining with CD31 and anti-α-SMA mAbs, and γH2AX positivity was assessed by enumeration of foci (Fig. 6 A, B). The mean percentage of γH2AX foci in EC from five AAA aortic tissue samples was 38% (range 22%–64%), while that in VSMC was 35% (range 28%–59%). In EC and VSMC from six control aortic tissues, mean percentages of γH2AX foci were 2% (range 0.1%–6%) and 1.7% (range 0.15%–5.7%), respectively. γH2AX-positive foci were significantly more abundant in EC and VSMC from AAA wall compared to controls (p = 0.006 and p = 0.010, respectively) (Fig. 6 C). These results indicate increased occurrence of DSBs in EC and VSMC from AAA *vs* normal aortic tissue, consistent with reduced TL and replicative senescence of these cell types.

### Vascular remodeling in aortic aneurysmatic wall

EC from patients with atherosclerosis, aorta manifests high rates of proliferation and increased cellular turnover that may contribute to age-dependent attrition of telomeres [48]. It has been hypothesized but never demonstrated that a similar mechanism operates in EC from AAA patients [49]. To test this hypothesis, tissue sections of aortic wall from AAA patients or controls were stained by immunofluorescence with an anti-Ki-67 mAb in combination with CD31 or anti α-SMA mAbs. Ki-67 represents an excellent marker of the growth fraction of a given cell population while is not expressed in resting cells [50]. The proportion of Ki-67^+^ EC from AAA patients was significantly higher than that detected in EC from control aorta (0.85±0.6 *versus* 0.1±0.2%; p = 0.005). Likewise, Ki-67^+^ VSMC were significantly more abundant in aortic tissue from AAA patients than controls (0.56±0.4 *versus* 0.1±0.2%; p = 0.005). These findings indicate that EC and VSMC from AAA patients contain a higher fraction of proliferating cells than their normal counterparts, conceivably indicative of increased vascular remodeling [49] in AAA.

### Oxidative DNA damage and telomere shortening in blood lymphocytes

**Figure 7:**
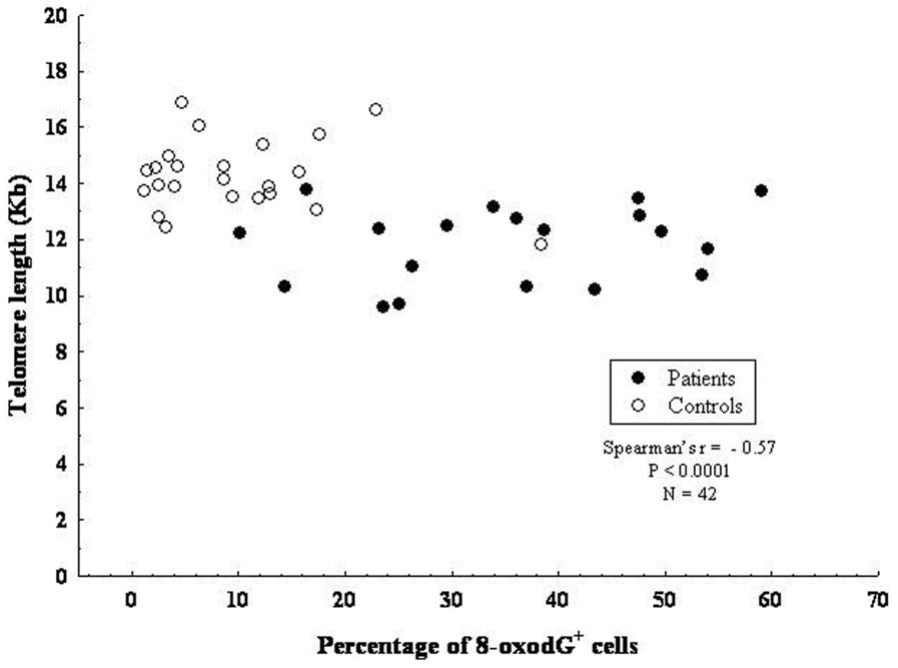
Relationship between telomere shortening and DNA damage in blood lymphocytes from AAA patients. Spearman's correlation test. Linear regression analysis between telomere length and accumulation of ROS-induced oxidative DNA damage, assessed by 8-oxo-dG staining, in blood lymphocytes from 19 AAA patients and from 23 controls. The Spearman's rank correlation coefficient (r_S_) is  = −0.57.

Blood lymphocytes are easily obtained from AAA patients and various studies [38], [39] including this indicate that analysis of TL in these cells provides a reliable estimate of vascular aging. We next correlated TL and extent of oxidative DNA damage in blood lymphocytes from AAA patients and found a moderate statistically significant inverse correlation (r_S_ = −0.57; p<0.0001) (Fig. 7).

## Discussion

AAA originates from the interaction of genetic predisposition with environmental factors that concur at weakening the wall of abdominal aorta and promote its progressive dilation [16]–[18]. Inflammation is not only associated with clinically overt AAA but may play also a role in disease pathogenesis [18], [27]–[31]. Aneurisms are often infiltrated with activated lymphocytes, macrophages and neutrophils that release matrix metalloproteases and serine proteases which degrade structural proteins of the aortic wall such as elastin, collagen and laminin [18], [27]–[31]. Furthermore, infiltrating cells release pro-inflammatory cytokines such as IL-6, osteopontin and the chemokine CCL-2 that recruit additional inflammatory cells [32].

Oxidative stress is always associated with inflammation and determines tissue damage due to increased production or impaired clearance of ROS. Previous studies have shown that markers of oxidative stress, e.g. inducible nitric oxide synthase, nitrotyrosine, nitrites, NADPH oxidase, and p22^phox^ are increased in the media and adventitia of human AAA tissues as compared to normal aorta. These findings have been reproduced in different animal models of human AAA [18], [25], [26], [33], [34].

Oxidative stress is also involved in telomere shortening. In this respect, it was shown recently that VSMC present in human arterial atherosclerotic plaques exhibited oxidative DNA damage and short telomeres, suggesting a potential relation between these findings [51]. The issue of oxidative DNA damage in EC and VSMC present in the AAA aortic wall has never been investigated before.

Interest in telomere research in AAA has been fueled by some studies showing that telomeres are shortened in AAA patients and that TL in circulating lymphocytes correlates with that in the aortic wall [38], [39].

In this study we investigated TL in different cell types from patients with AAA and matched controls including EC and VSMC from the aortic wall, epidermal cells and blood lymphocytes using Q-FISH [40]–[43]. This technique allowed us to estimate accurately TL in each individual cell type and to perform correlative analyses. Telomeres were found to be significantly shortened in EC, VSMC, epidermal cells and blood lymphocytes from AAA patients compared to matched controls, indicating that AAA is a systemic disorder involving different cell types in different anatomical locations and conceivably related to genetic predisposition [22]–[24] and/or life style [16]–[18].

We next investigated the occurrence of oxidative DNA damage in EC and VSMC, as well as in epidermal cells and blood lymphocytes, from AAA patients using immunofluorescence for 8-oxo-dG. Oxidative damage to telomeric DNA provoked by ROS [45] results in the formation of 8-oxo-dG which contributes to impair physiological maintenance of TL *in vitro* [14], [15], [44]. Recent data indicate that triplet guanines present in telomeric TTAGGG repeats contribute to the preferential accumulation of oxidative base damage in telomeres *in vivo* [15]. 8-oxo-dG in some telomere structures (e.g., fork-opening, 3′-overhang, and D-loop) are less effectively removed and the presence of 8-oxo-dG can lead to telomere shortening accompanied by strand breakage [44].

A strong 8-oxo-dG immunoreactivity was detected in EC and VSMC, as well as in epidermal cells and blood lymphocytes from AAA patients, with the highest percentage of positive cells found in EC and VSMC. This latter observation is consistent with a previous report showing a close association between infiltrating macrophages and the extent of oxidative DNA damage in the aortic wall from AAA patients [52], [53]. Moreover, we demonstrated that EC and VSMC from aortic aneurysmatic wall expressed γH2AX histone foci at high level, indicative of DNA DSB concentrating preferentially in telomeres in a high oxidative environment [46], [47].

The results obtained in this study suggest that oxidative stress, like telomere shortening, is a systemic phenomenon in AAA patients. Examples of other systemic disorders characterized by the occurrence of oxidative stress in multiple cell types are psoriasis and scleroderma [54].

Recent studies indicate that shortening of telomeres may promote oxidative stress associated with impaired mitochondrial biogenesis and function, decreased gluconeogenesis and cardiomyopathy [55]–[57]. Thus, the cause and effect relation between oxidative stress and telomere shortening is still debated.

Oxidative stress promotes EC and VSMC apoptosis [33], [34]. The loss of VSMC, which represent the major sources of structural proteins of the extracellular matrix, and the consequent disorganization of the latter stimulate aneurysm remodeling [33], [34]. With this background, we investigated expression of the proliferation marker Ki-67 in VSMC from AAA patients and controls as potential indicator of aneurysm remodeling and found that Ki-67^+^ VSMC from AAA patients were indeed significantly increased. Moreover, the percentage of Ki67^+^ EC was significantly higher in aortic wall from AAA patients, suggesting that the process of aneurysm remodeling may involve not only VSMC but also EC.

Blood lymphocytes are an attractive cell fraction for the investigation of TL and, as shown here for the first time, of oxidative DNA damage in AAA patients. We therefore asked whether there was an inverse correlation between TL and accumulation of ROS-induced oxidative DNA damage, as assessed by 8-oxo-dG staining, in blood lymphocytes from AAA patients. We detected a moderate inverse correlation between TL and oxidative DNA damage, suggesting the involvement of additional factors (e.g. genetic, environmental) in telomere shortening. It must be stressed that neither telomere shortening nor increased oxidative DNA damage are specifically associated with AAA pathogenesis, but rather represent hallmarks of a wide spectrum of inflammatory and degenerative disorders [8], [18], [32].

It has been reported that anti-oxidant drugs such as vitamin E can reduce the size of AAA as well as the incidence of aortic rupture in pre-clinical experimental models [58]. Furthermore, angiotensin II type I receptor blockers or statin were found to suppress significantly the expression of p22^phox^ in the aortic wall of patients with thoracic aorta aneurysm [36]. Based upon our results, we propose that the biological activity of these and other emerging therapeutic strategies for AAA are monitored through the simple investigation of TL in circulating lymphocytes by Q-FISH. This latter technique is easily performed by experienced personnel and requires small aliquots of cells, as opposed to Southern blot [38], [39] that is cumbersome and time-consuming, and requires large numbers of cells. Follow-up of this proposal in future studies is warranted.

## Materials and Methods

### Study design and tissue samples

Patients and controls were matched not only for age, but also for absence of neoplasms, infections and chronic inflammation, chronic obstructive pulmonary disease, diabetes mellitus, nephropathy, liver disease, symptomatic obstructive coronary, cerebrovascular and peripheral diseases, and smoking habits. Twenty-six % of AAA patients had positive familial history for abdominal aortic aneurism. Fifty seven subjects were enrolled for TL studies (Table 1), i.e. twenty three AAA patients whose mean age was 71.3 (range 61–78 years) and 34 apparently healthy control subjects whose mean age was 68.7 (range 62–77 years). Blood lymphocytes were obtained from peripheral venous blood sample of the superficial vein of the arm. Skin biopsies were obtained from eleven AAA patients (mean age 70, range 76–65) at the time of aortic surgery and from six control subjects (mean age 69, range 73–65) at the time of blood sampling by a poorly invasive skin biopsy technique. Aortic tissue samples were obtained from the aneurismal sac in twenty one of the above mentioned patients. The processing of these samples was carried out by the Genoa Vascular Bio-Bank operated by two of us (DP and FD), which collects and stores for research purposes samples of atherothrombotic and aneurysmatic arterial wall obtained intra-operatively, following strictly standard operating procedures and ethical regulations. Twenty specimens of control abdominal aortic tissue were obtained from the Institute of Forensic Medicine of the University of Genoa (mean age 68.7 years, range 60–81 years). These samples were from individuals who died of accidental trauma or suicide and had no autoptic evidence of aortic aneurism or other medical conditions that precluded recruitment to the study.

Participation in the study of AAA patients and control subjects was based upon informed consent of patients or legal representatives. The study conformed to the principles of the Helsinki declaration and was approved by the Ethics Committee of the Azienda Ospedale-Università S. Martino, Genoa, Italy.

### Sample Preparation

Mononuclear cells from heparinized peripheral blood were isolated by Histopaque-1077 (Sigma, St. Louis, MO) gradient centrifugation and washed three times in phosphate buffered saline (PBS). Cytospin preparations of peripheral blood lymphocytes from AAA patients and controls were cytocentrifuged and fixed in 4% paraformaldehyde in PBS. In the operating room, AAA tissues were rapidly washed in physiological saline. Thereafter they were fixed in 4% phosphate-buffered formaldehyde, processed into paraffin blocks, and subjected to sectioning. Formalin-fixed, paraffin-embedded normal aorta tissue blocks were obtained from the University of Genoa Bio-Bank and processed as above.

### Telomere length measurement by quantitative fluorescence *in situ* hybridization (Q-FISH) in interphase nuclei

Quantitative fluorescence *in situ* hybridization (Q-FISH) of telomeres has been extensively used to obtain quantitative information on TL distributions [40]–[43]. In addition, the low detection limit of Q-FISH (0.1 kb of telomere repeats) allows quantification of critically short telomeres that go undetected by Southern blot analysis. Interphase Q-FISH method, which builds on conventional Q-FISH, combines labeling of telomeres in interphase nuclei, using a fluorescent peptide nucleic acid (PNA) probe against telomeric repeats, with automated microscopy. The PNA probe for telomeric sequences is a ready-to-use probe included in the Telomere PNA FISH Kit/Cy3 (Dako Cytomation, Hamburg, Germany). The hybridization was performed according to the manufacturer's instructions. Slides were mounted with antifade solution (Vector Laboratories, Burlingame, CA). Slide scanning, cell identification, intensity measurement, and quantification of TL were performed using the fluorescence-based microscopic scanning system E-1000 Nikon (Nikon, Japan) and a high-resolution CCD camera. For TL quantification, we measured Cy3 intensity in single nuclei. The slide scanning and cell analysis procedures were performed using a 100× Nikon objective. The Cy3 pan-telomeric probe intensity was measured by an appropriate filter. Telomere fluorescence signals were quantified by using the Genikon program (Nikon). A minimum of 20 nuclei were scanned for every sample and the mean value of the fluorescence ratios of all cells analyzed was calculated.

### Calibration of quantitative FISH analysis

To validate the use of Q-FISH for measurements of the length of telomeric repeats we hybridized five cell lines with different size of TTAGGG repeat sequences with the Cy3-PNA telomeric probe. Telomere fluorescence units (TFU) are converted into kilobase (Kb) by external calibration with the L5178Y-S and L5178Y-R murine lymphoma cell lines, MEF murine embryonic fibroblast cell line, MCF7 and HeLa human tumor cell lines with known TL of 10.2 Kb, 79.7 kb, 47 Kb, 4.07 Kb and 3.44 Kb, respectively [42]. For each cell line the median fluorescence intensity was directly proportional to the size of the TTAGGG repeats sequences and the resulting calibration line was used to express telomere fluorescence in TFU with corresponding Kb of TTAGGG repeats. The resulting calibration line was used to transform arbitrary TFU (X) into TL in Kb (Y) by means of the formula Y = −16.1546+287.2233×X (R = 0.997) (Fig. 1).

### Immunofluorescence studies

Indirect immunofluorescence was performed on 4-µm-thick formalin-fixed, paraffin embedded tissue sections or cytospins as previously described [59]. The following primary monoclonal antibodies were used: anti-human(h)CD31 (diluted 1/50; Dako Cytomation, Hamburg, Germany), anti-α-smooth muscle actin (1/60; α-SMA; Dako), anti-human Ki-67 (1/60; Dako), anti-8-oxo-dG (1/100; Santa Cruz Biotechnology, Europe), anti-γ-H2AX (1/50; Millipore). After antigen retrieval slides were incubated with primary antibodies overnight at 4°C, followed by AlexaFluor-488 or 568-conjugated anti-mouse immunoglobulin G (1/200 dilution, Molecular Probes, Eugene, OR, US).

### Statistical analysis

Because observed TL had a skewed distribution, the statistical analyses were performed on natural log-transformed data. Standard linear regression techniques were used to associate TL with individual factors and to adjust for age (in years) and gender. Descriptive statistics were firstly performed; absolute frequencies and percentages were reported for qualitative data, medians with quartiles (1st–3rd q) were reported for quantitative variables. Comparison of categorical variables between cases and controls was made by the chi-square test or by the Fisher's Exact test in case of expected frequencies <5. Comparison of quantitative variables between cases and controls was made by the Mann-Whitney U test. Correlations were evaluated by the Spearman's rank correlation coefficient (rS); rS values from 0.40 to 0.59 were considered moderate, from 0.60 to 0.79 were considered strong, and from 0.80 to 1 were considered very strong; values<0.4 were considered weak [60]. All statistical test were two-sided and a P value<0.05 was considered as statistically significant. The software “Statistica”, release 8 (StatSoft Co., Tulsa, OK) was used to perform all the analyses.
